# TyG Index and Obesity‐Related Measures in Relation to All‐Cause Mortality Among HSV‐Positive Adults

**DOI:** 10.1155/ije/1608622

**Published:** 2026-02-26

**Authors:** Jun Wei, Jun Zhang, Yang Liu

**Affiliations:** ^1^ School of Basic Medical Sciences, Jilin Medical University, Jilin, China, jlu.edu.cn; ^2^ Biomedical Sciences, Edinburgh Medical School, College of Medicine and Veterinary Medicine, The University of Edinburgh, Edinburgh, UK, ed.ac.uk; ^3^ Zhejiang University School of Medicine, Zhejiang University–University of Edinburgh Institute, Haining, China, zju.edu.cn

**Keywords:** all-cause mortality, cardiovascular mortality, cohort study, herpes simplex virus, triglyceride–glucose index

## Abstract

**Background:**

Previous studies have extensively explored the association between the triglyceride–glucose (TyG) index and mortality; however, evidence specific to the herpes simplex virus (HSV)–positive population remains limited. Therefore, this study aims to investigate the association between the TyG index and mortality among HSV‐positive adults, providing new insights into this field.

**Methods:**

This study included 8465 HSV‐positive adults from the National Health and Nutrition Examination Survey (NHANES) 1999–2018, with mortality follow‐up through 2019. The TyG index and four derived indices, TyG‐BMI, TyG‐WC, TyG‐WHtR, and TyG‐ABSI, were calculated using fasting triglycerides, glucose, and anthropometric data. Associations with all‐cause mortality were assessed using Cox proportional hazards models. Two‐piecewise Cox regression models were applied to explore potential threshold effects.

**Results:**

Over a median follow‐up of 143 months, 297 all‐cause deaths (3.5%) occurred. Higher levels of the TyG index and its derivatives were significantly associated with increased all‐cause mortality. TyG‐WHtR showed the strongest association (HR: 2.08, 95% CI: 1.41–3.06), followed by TyG‐WC (HR: 1.95, 95% CI: 1.34–2.82), TyG‐BMI (HR: 1.22, 95% CI: 1.11–1.33), and the TyG index itself (HR: 1.35, 95% CI: 1.20–1.52). TyG‐ABSI also demonstrated a positive linear association (HR: 1.33, 95% CI: 1.18–1.50).

**Conclusions:**

Elevated levels of the TyG index and its obesity‐related derivatives, particularly TyG‐WHtR, were associated with increased all‐cause mortality in HSV‐positive adults.

## 1. Introduction

The herpes simplex virus (HSV), which comprises HSV‐1 and HSV‐2, is categorized as a double‐stranded enveloped DNA virus featuring a tegument layer situated between its envelope and capsid [1]. Globally, HSV infections are prevalent, affecting approximately 67% of people under 50 years of age with HSV‐1 and 13% of individuals aged 15 to 49 with HSV‐2 [2]. While HSV‐1 is known to result in common cold sores and herpetic keratitis, it can also lead to rare yet fatal cases of encephalitis. On the other hand, HSV‐2 is primarily responsible for genital herpes, and both types are significant contributors to neonatal herpes infections [3].

Insulin resistance (IR) refers to a condition in which the body shows reduced responsiveness to insulin, leading to a decreased ability of peripheral tissues to take up glucose and a reduced suppressive effect on glucose production in the liver, ultimately resulting in elevated blood sugar levels [4]. The triglyceride–glucose (TyG) index has become a significant measure for evaluating IR, providing a potentially more dependable assessment than conventional methods like the homeostasis model assessment of IR (HOMA‐IR) [5, 6]. In addition to the TyG index itself, several derived metrics, including TyG combined with waist circumference (TyG‐WC), TyG coupled with body mass index (TyG‐BMI), TyG alongside waist‐to‐height ratio (TyG‐WHtR), and TyG with a body shape index (TyG‐ABSI) [7, 8], are increasingly recognized as alternative measures of IR within a variety of metabolic disorders [9–11]. Recent studies have demonstrated that TyG‐related indices are significantly associated with cardiometabolic outcomes, including diabetes, cardiovascular disease (CVD), and increased risks of all‐cause and cardiovascular mortality, particularly in populations with metabolic dysfunction such as metabolic‐associated fatty liver disease (MASLD), IR, or cardiovascular–kidney–metabolic syndrome [12–14]. However, the relationship between TyG indices, HSV, and mortality remains underexplored.

Importantly, HSV infection represents a unique biological context for investigating metabolic risk. HSV establishes lifelong latent infection with intermittent reactivation [15], which has been associated with chronic low‐grade inflammation, immune dysregulation, and endothelial dysfunction [16]. Emerging evidence suggests that persistent viral infections may be associated with IR and metabolic disturbances, possibly via inflammatory and neuroendocrine mechanisms [17]. Unlike the general population, HSV‐positive individuals constitute a large but often clinically overlooked subgroup, in whom metabolic risk may accumulate silently at younger ages [18]. Therefore, evaluating TyG‐related indices in this infection‐defined population is of particular importance.

This study aimed to examine the associations between the TyG index, its derived indices, and all‐cause mortality in adults with HSV infection, using data from a nationally representative cohort. Understanding these associations may provide population‐level insights into metabolic health disparities among HSV‐positive individuals.

## 2. Methods

### 2.1. Study Design and Population

**FIGURE 1:**
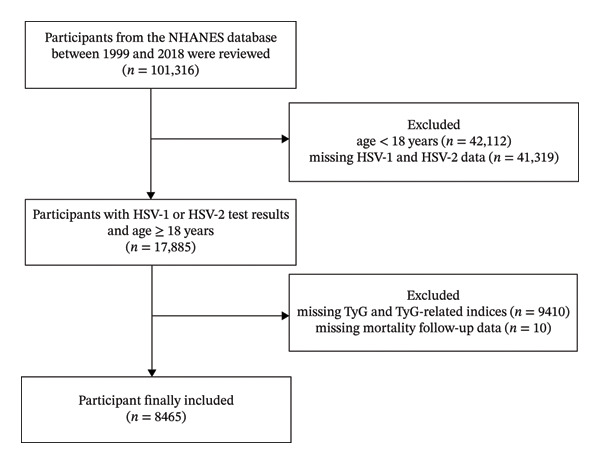
Flowchart of participant selection from the NHANES 1999–2018 database.

We used publicly available data from the National Health and Nutrition Examination Survey (NHANES) 1999–2018, a nationally representative series of cross‐sectional surveys with linked prospective mortality follow‐up via the National Death Index (NDI). Baseline data on demographics, lifestyle, health conditions, and laboratory measurements were obtained through standardized procedures. Participants aged ≥ 18 years with serologic evidence of HSV‐1 or HSV‐2 infection and valid fasting triglyceride and glucose measurements were eligible. After excluding individuals with missing TyG components or key covariates, the final analytic sample comprised 8465 HSV‐positive adults. The baseline examination year was defined by the survey cycle in which each participant was enrolled. Follow‐up time was calculated from the examination date until death or December 31, 2019. The median duration of follow‐up was 143 months (Figure 1).

### 2.2. Measurement of HSV

Blood samples were collected via venipuncture at a mobile examination center and processed according to standardized protocols. Serostatus for HSV‐1 and HSV‐2 was determined at Emory University using a type‐specific enzyme‐linked immunosorbent assay (ELISA) based on purified glycoproteins gG‐1 and gG‐2, which are unique to HSV‐1 and HSV‐2, respectively. These glycoproteins were immobilized on nitrocellulose membranes to allow differentiation between the two virus types, despite potential cross‐reactivity. Serum antibodies binding to gG‐1 or gG‐2 were detected using peroxidase‐conjugated goat antihuman IgG and a chromogenic substrate. A visible blue dot indicated seropositivity: Reactivity to gG‐1 signified prior HSV‐1 infection, while reactivity to gG‐2 indicated prior HSV‐2 infection. All participants provided written informed consent for the use of their samples in the study [19].

### 2.3. Exposure Assessment: TyG Index and Derived Indices

Fasting triglyceride (mg/dL) and fasting plasma glucose (mg/dL) levels were obtained from standardized laboratory assays in NHANES. The TyG index was calculated using the established formula:

TyG = ln [fasting triglyceride (mg/dL) × fasting glucose (mg/dL)/2].

In addition to the TyG index, we derived four composite indices to incorporate both metabolic and anthropometric information:

TyG‐BMI = TyG × body mass index (BMI, kg/m^2^).

TyG‐WC = TyG × waist circumference (WC, cm).

TyG‐WHtR = TyG × waist‐to‐height ratio (WHtR).

TyG‐ABSI = TyG × ABSI, where ABSI = WC (m)/[BMI^(2/3) × height^(1/2)]. All indices were treated as continuous variables in the primary analyses. For descriptive and secondary analyses, each index was also categorized into tertiles based on their distribution in the analytic sample.

### 2.4. Outcome Ascertainment

Mortality status and cause of death were determined by linkage to the NDI through December 31, 2019. The primary outcome was all‐cause mortality, and the secondary outcome was CVD mortality, identified using ICD‐10 codes I00–I09, I11, I13, and I20–I51. Follow‐up time was defined as the interval from the date of the NHANES examination to the date of death or the end of follow‐up, whichever occurred first.

### 2.5. Covariates

Covariates were selected a priori based on their potential to confound the association between TyG‐related indices and mortality. Demographic characteristics included age (years), sex (male/female), race/ethnicity (Non‐Hispanic White, Non‐Hispanic Black, Mexican American, Other Hispanic, or Other Race), marital status (married/living with partner, widowed/divorced/separated, or never married), and poverty income ratio categorized as poor, nearly poor, middle income, or high income. Educational attainment was classified as below high school, high school graduate, or above high school. Health‐related behaviors included smoking status (never, former, or current), alcohol consumption (never, former, mild, moderate, or heavy), and physical activity, quantified as total weekly metabolic equivalent of task (MET) minutes and dichotomized as ≥ 0600 versus < 600 MET min/week according to WHO guidelines. Clinical covariates included BMI (kg/m^2^), hypertension (self‐reported or measured BP ≥ 140/90 mmHg), and diabetes mellitus (defined by self‐report, fasting glucose  ≥ 126 mg/dL, 2 h OGTT ≥ 200 mg/dL, or HbA1c ≥ 6.5%). Although dyslipidemia and CVD history were collected, they were not included in the final multivariable models due to potential collinearity and risk of overadjustment, respectively. All covariates were measured at baseline during the NHANES examination.

### 2.6. Statistical Analysis

All analyses were conducted following a predefined analytical plan to evaluate the associations between the TyG index and its obesity‐related derivatives (TyG‐BMI, TyG‐WC, TyG‐WHtR, and TyG‐ABSI) with all‐cause and cardiovascular mortality among HSV‐positive adults. Baseline characteristics were summarized using means ± standard deviations for continuous variables and proportions for categorical variables, and differences by TyG tertiles were compared using one‐way ANOVA or chi‐square tests. To explore potential nonlinear relationships between TyG‐related indices and mortality outcomes, smooth curve fitting was performed using generalized additive models (GAMs) with penalized splines [20]. If a nonlinear pattern was suggested, two‐piecewise Cox proportional hazards models were applied to identify potential threshold effects [21, 22]. Inflection points were determined by selecting the value with the optimal model fit, and statistical significance was assessed by comparing the log‐likelihoods of the one‐piece versus two‐piece models. The model was adjusted for age (years), sex, ethnicity, marital status, poverty income ratio, education level, smoking status, alcohol use, and total physical activity (MET/week). Additionally, Kaplan–Meier survival curves stratified by TyG tertiles were generated [23]. All statistical analyses were performed using R software, Version 4.3.2 (R Foundation for Statistical Computing, Vienna, Austria) and EmpowerStats, Version 5.2 (X & Y Solutions Inc., Boston, MA, USA). A two‐sided *p* value < 0.05 was considered statistically significant.

## 3. Results

### 3.1. Demographic Characteristics of the HSV‐Positive Population

During the study period, a total of 8465 participants with serological evidence of HSV Type 1 or Type 2 infection from the NHANES 1999–2018 cycles were included in the analysis (Table 1). The mean age of the study population was 33.87 years, and females accounted for a slightly higher proportion than males (4693 vs. 3772). However, male participants showed a higher risk of all‐cause mortality compared to females (57.24% vs. 42.76%). The majority of participants were Non‐Hispanic White (32.14%), followed by Non‐Hispanic Black (23.97%) and Mexican American (26.30%). Regarding education, 45.76% of participants had completed high school, while 44.41% had education above high school. More than half (56.86%) were married or living with a partner. At baseline, 24.73% were current smokers, and 24.70% reported heavy alcohol use. The mean BMI was 28.67 kg/m^2^, and the mean values for TyG and its derived indices were as follows: TyG 8.54, TyG‐BMI 246.01, TyG‐WC 821.05, TyG‐WHtR 4.91, and TyG‐ABSI 0.68. A total of 297 deaths were recorded during follow‐up. Compared to survivors, nonsurvivors were older, more likely to be male and Non‐Hispanic White, and had lower education and income levels. They also had higher mean values of height, weight, WC, WHtR, ABSI, triglycerides, fasting glucose, and all TyG‐related indices (all *p* <  0.001), and were more likely to be widowed/divorced/separated. Furthermore, nonsurvivors showed higher prevalence of smoking, former alcohol use, physical inactivity (< 600 MET/week), hypertension, diabetes, CVD, and hyperlipidemia.

**TABLE 1:** The demographic characteristics of the HSV‐positive population in the present study were stratified by survival status.

Variable	Total (*n* = 8465)	Survivor (*n* = 8168)	Nonsurvivors (*n* = 297)	*p* value
Age (years)	33.87 ± 9.45	33.70 ± 9.42	38.57 ± 9.20	< 0.001
Sex				< 0.001
Male	3772 (44.56%)	3602 (44.10%)	170 (57.24%)	
Female	4693 (55.44%)	4566 (55.90%)	127 (42.76%)	
Height (cm)	167.54 ± 9.82	167.44 ± 9.83	170.40 ± 9.23	< 0.001
Weight (kg)	80.70 ± 21.72	80.52 ± 21.62	85.79 ± 23.99	< 0.001
BMI (kg/m^2^)	28.67 ± 7.06	28.64 ± 7.02	29.55 ± 7.98	0.031
Waist (cm)	95.69 ± 16.35	95.55 ± 16.26	99.53 ± 18.43	< 0.001
Waist‐to‐height ratio	0.57 ± 0.10	0.57 ± 0.10	0.59 ± 0.11	0.020
Triglyceride (mg/dL)	129.16 ± 121.49	128.23 ± 121.59	154.69 ± 116.07	< 0.001
Glucose (mg/dL)	5.55 ± 1.71	5.52 ± 1.61	6.33 ± 3.38	< 0.001
TyG	8.54 ± 0.68	8.53 ± 0.67	8.81 ± 0.77	< 0.001
TyG‐BMI	246.01 ± 67.83	245.44 ± 67.24	262.01 ± 80.98	< 0.001
TyG‐WC	821.05 ± 175.84	818.84 ± 174.20	882.00 ± 207.61	< 0.001
TyG‐WHTR	4.91 ± 1.04	4.90 ± 1.03	5.19 ± 1.23	< 0.001
TyG‐ABSI	0.68 ± 0.08	0.68 ± 0.08	0.71 ± 0.08	< 0.001
Ethnicity				< 0.001
Non‐Hispanic White	2721 (32.14%)	2593 (31.75%)	128 (43.10%)	
Non‐Hispanic Black	2029 (23.97%)	1931 (23.64%)	98 (33.00%)	
Mexican American	2226 (26.30%)	2174 (26.62%)	52 (17.51%)	
Other Hispanic	791 (9.34%)	783 (9.59%)	8 (2.69%)	
Other race	698 (8.25%)	687 (8.41%)	11 (3.70%)	
Marital status, *n* (%)				0.004
Married/living with partner	4813 (56.86%)	4654 (56.98%)	159 (53.54%)	
Widowed/divorced/separated	1028 (12.14%)	972 (11.90%)	56 (18.86%)	
Never married	2242 (26.49%)	2170 (26.57%)	72 (24.24%)	
Missing	382 (4.51%)	372 (4.55%)	10 (3.37%)	
Poverty income ratio				0.034
Poor	2164 (25.56%)	2077 (25.43%)	87 (29.29%)	
Nearly poor	2142 (25.30%)	2060 (25.22%)	82 (27.61%)	
Middle income	1953 (23.07%)	1881 (23.03%)	72 (24.24%)	
High income	1549 (18.30%)	1515 (18.55%)	34 (11.45%)	
Missing	657 (7.76%)	635 (7.77%)	22 (7.41%)	
Education level, *n* (%)				0.004
Below high school	825 (9.75%)	790 (9.67%)	35 (11.78%)	
High school	3874 (45.76%)	3717 (45.51%)	157 (52.86%)	
Above high school	3759 (44.41%)	3655 (44.75%)	104 (35.02%)	
Missing	7 (0.08%)	6 (0.07%)	1 (0.34%)	
Smoking status, *n* (%)				
Never	4488 (53.02%)	4396 (53.82%)	92 (30.98%)	
Former	1190 (14.06%)	1148 (14.05%)	42 (14.14%)	
Now	2093 (24.73%)	1947 (23.84%)	146 (49.16%)	
Missing	694 (8.20%)	677 (8.29%)	17 (5.72%)	
Alcohol use, *n* (%)				< 0.001
Never	980 (11.58%)	951 (11.64%)	29 (9.76%)	
Former	932 (11.01%)	876 (10.72%)	56 (18.86%)	
Mild	1841 (21.75%)	1789 (21.90%)	52 (17.51%)	
Moderate	1193 (14.09%)	1149 (14.07%)	44 (14.81%)	
Heavy	2091 (24.70%)	2007 (24.57%)	84 (28.28%)	
Missing	1428 (16.87%)	1396 (17.09%)	32 (10.77%)	
Total physical activity (MET/week)				< 0.001
< 600	2366 (27.95%)	2259 (27.66%)	107 (36.03%)	
≥ 600	4103 (48.47%)	4003 (49.01%)	100 (33.67%)	
Missing	1996 (23.58%)	1906 (23.33%)	90 (30.30%)	
Hypertension				< 0.001
No	6727 (79.50%)	6558 (80.32%)	169 (56.90%)	
Yes	1735 (20.50%)	1607 (19.68%)	128 (43.10%)	
Diabetes mellitus				< 0.001
No	6436 (81.12%)	6237 (81.51%)	199 (70.57%)	
Yes	622 (7.84%)	572 (7.48%)	50 (17.73%)	
IFG	477 (6.01%)	452 (5.91%)	25 (8.87%)	
IGT	399 (5.03%)	391 (5.11%)	8 (2.84%)	
Hyperlipidemia				0.016
No	2979 (35.19%)	2894 (35.43%)	85 (28.62%)	
Yes	5486 (64.81%)	5274 (64.57%)	212 (71.38%)	
Cardiovascular diseases				< 0.001
No	7460 (97.24%)	7209 (97.52%)	251 (89.64%)	
Yes	212 (2.76%)	183 (2.48%)	29 (10.36%)	

*Note:* TyG, triglyceride–glucose index; WHtR, waist‐to‐height ratio.

Abbreviations: BMI, body mass index; HDL‐C, high‐density lipoprotein cholesterol; IFG, impaired fasting glycemia; IGT, impaired glucose tolerance; LDL‐C, low‐density lipoprotein cholesterol; MET, metabolic equivalent of task; WC, waist circumference.

### 3.2. Baseline Characteristics of Participants According to TyG Index Tertiles

A total of 8465 participants were categorized into tertiles based on the TyG index: low (*n* = 2818), middle (*n* = 2823), and high (*n* = 2824). As the TyG index increased, participants were more likely to be older and male. Anthropometric indicators, including height, weight, BMI, WC, WHtR, and ABSI, showed increasing trends across TyG tertiles. Biochemical parameters such as triglyceride and fasting glucose levels also increased progressively, along with derived indices including TyG‐BMI, TyG‐WC, TyG‐WHtR, and TyG‐ABSI. Regarding ethnicity, the proportions of Non‐Hispanic White and Mexican American participants were higher in the high TyG group, while the proportion of Non‐Hispanic Black participants was lower. Participants in the highest tertile were more often married or living with a partner and had lower educational attainment. Lifestyle characteristics also varied significantly: Higher TyG tertiles were associated with greater proportions of current and former smokers and increased former and heavy alcohol consumption. Moreover, participants in the high TyG group were less likely to achieve ≥ 600 MET/week of physical activity. Clinically, the prevalence of hypertension, diabetes mellitus, impaired fasting glucose, impaired glucose tolerance, hyperlipidemia, and CVDs was higher in the upper tertiles (all *p* < 0.001). No statistically significant differences were observed in poverty income ratio across TyG groups (*p* = 0.295).

### 3.3. Nonlinear Associations Between TyG‐Related Indices and All‐Cause Mortality

To examine the potential nonlinear relationships between TyG‐related indices and all‐cause mortality, smooth curve fitting and threshold effect analysis were applied. As shown in Figures 2(a), 2(b), 2(c), 2(d), 2(e), most indices demonstrated U‐shaped or J‐shaped associations with mortality risk, indicating possible nonlinear effects. Inflection points were identified through two‐piecewise linear regression models, with detailed estimates presented in Table 2.

FIGURE 2Smooth curve fitting of the association between TyG‐related indices and all‐cause mortality. Plots display the dose–response relationships of all‐cause mortality risk with (a) TyG index, (b) TyG‐BMI index, (c) TyG‐WC index, (d) TyG‐WHtR index, and (e) TyG‐ABSI index. The solid red lines represent the estimated log‐relative risk based on smooth curve fitting, while the light blue shaded areas indicate 95% confidence intervals. The model was adjusted for age (years), sex, ethnicity, marital status, poverty income ratio, education level, smoking status, alcohol use, and total physical activity (MET/week). Abbreviations: ABSI, a body shape index; BMI, body mass index; TyG, triglyceride–glucose index; WC, waist circumference; WHtR, waist‐to‐height ratio.

**Figure:**
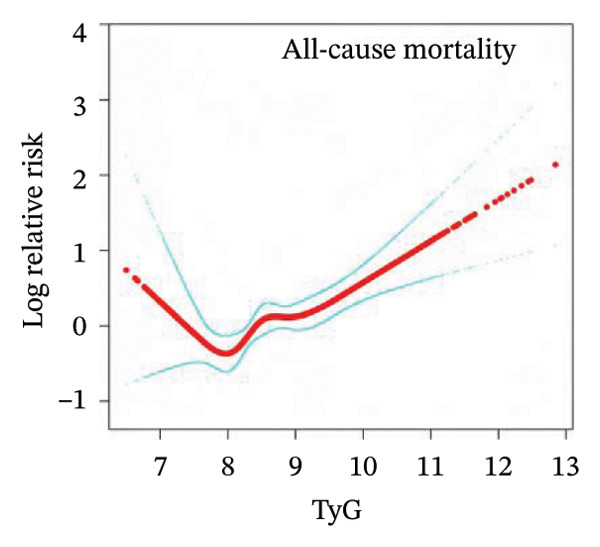
(a)

**Figure:**
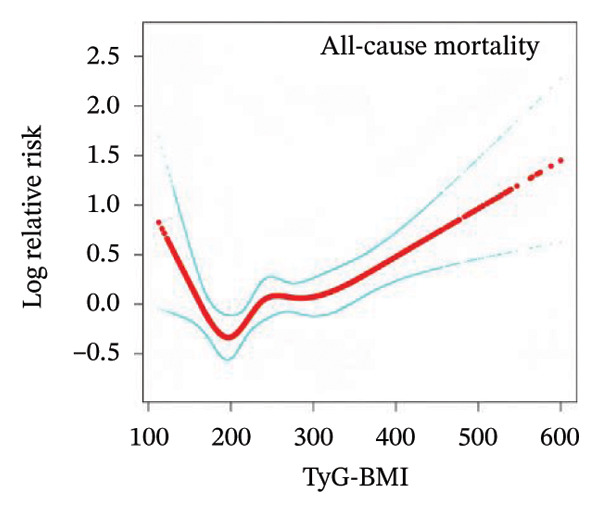
(b)

**Figure:**
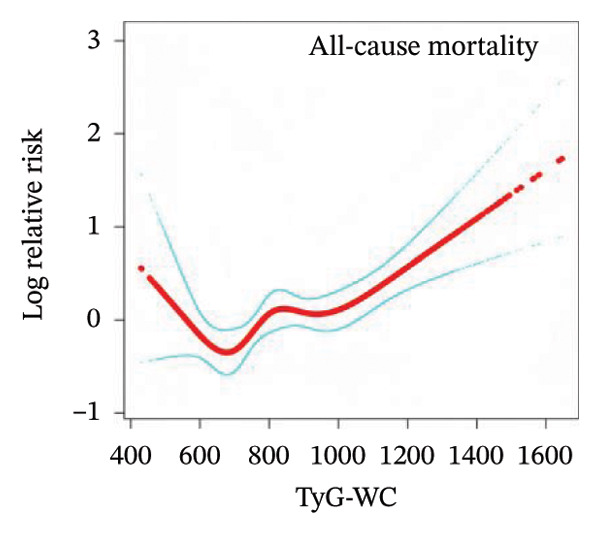
(c)

**Figure:**
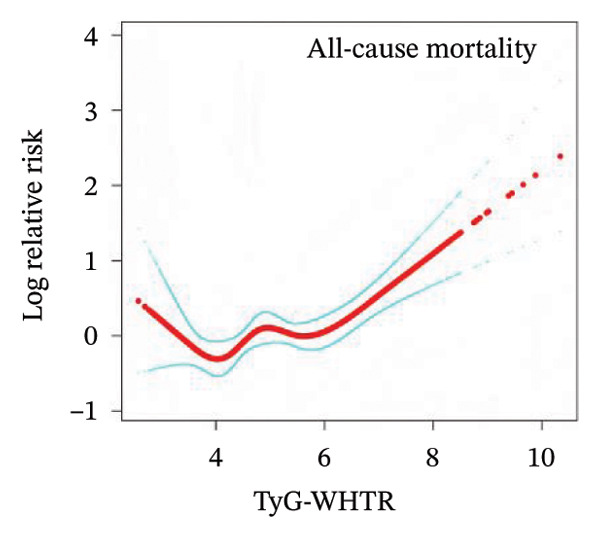
(d)

**Figure:**
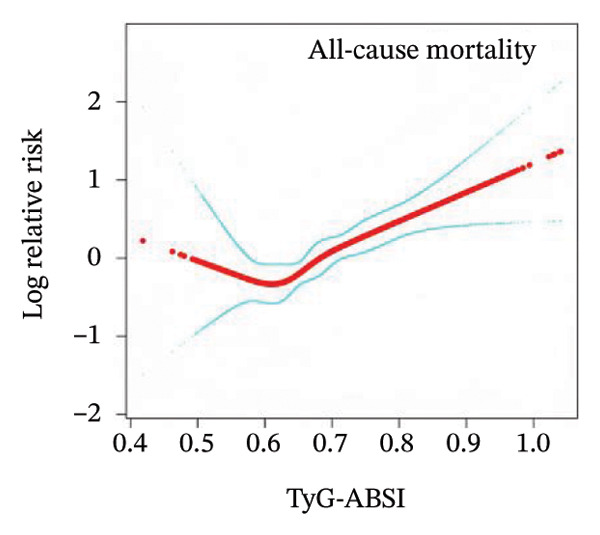
(e)

**TABLE 2:** Analysis of the threshold effect of TyG and TyG‐related indices on all‐cause mortality.

	Adjusted HR (95% CI)	*p* value
*All-cause mortality*		
TyG (per SD change)		
Linear model	1.30 (1.16, 1.45)	< 0.0001
Inflection point	TyG = 7.902	
TyG < 7.902	0.69 (0.33, 1.43)	0.3225
TyG > 7.902	1.35 (1.20, 1.52)	< 0.0001
P for log‐likelihood ratio	**0.112**	

*TyG-BMI (per SD change)*		
Linear model	1.18 (1.07, 1.30)	0.0006
Inflection point	TyG‐BMI = 157.424	
TyG‐BMI < 157.424	0.02 (0.00, 0.19)	0.0004
TyG‐BMI > 157.424	1.22 (1.11, 1.33)	< 0.0001
P for log‐likelihood ratio	**0.002**	

*TyG-WC (per SD change)*		
Linear model	1.27 (1.13, 1.42)	< 0.0001
Inflection point	TyG‐WC = 1140.533	
TyG‐WC < 1140.533	1.15 (1.00, 1.33)	0.0427
TyG‐WC > 1140.533	1.95 (1.34, 2.82)	0.0004
P for log‐likelihood ratio	**0.030**	

*TyG-WHTR (per SD change)*		
Linear model	1.27 (1.13, 1.43)	< 0.0001
Inflection point	TyG‐WHTR = 6.799	
TyG‐WHTR < 6.799	1.16 (1.01, 1.33)	0.0401
TyG‐WHTR > 6.799	2.08 (1.41, 3.06)	0.0002
P for log‐likelihood ratio	**0.020**	

*TyG-ABSI (per SD change)*		
Linear model	1.33 (1.18, 1.50)	< 0.0001
Inflection point	TyG‐ABSI = 0.63	
TyG‐ABSI < 0.63	0.93 (0.55, 1.57)	0.7901
TyG‐ABSI > 0.63	1.39 (1.22, 1.58)	< 0.0001
P for log‐likelihood ratio	**0.185**	

*Note:* Hazard ratios (HRs) are reported per one‐standard deviation (SD) increase in each TyG‐related index to improve comparability across measures with different units and scales. One SD corresponds to 0.678 for the TyG index, 68.565 for TyG‐BMI, 175.845 for TyG‐WC, 1.043 for TyG‐WHtR, and 0.076 for TyG‐ABSI. Bold font indicates that the log‐likelihood ratio test is statistically significant (*p* < 0.05). The model was adjusted for age (years), sex, ethnicity, marital status, poverty income ratio, education level, smoking status, alcohol use, and total physical activity (MET/week); TyG, triglyceride–glucose index; WHtR, waist‐to‐height ratio. ABSI, a body shape index.

Abbreviations: BMI, body mass index; CI, confidence interval; HR, hazard ratio; WC, waist circumference.

For the TyG index (Figure 2(a)), a U‐shaped association with mortality was observed. The estimated inflection point was 7.902 (Table 2). Below this threshold, the association was not statistically significant (HR = 0.69, 95% CI: 0.33–1.43, *p* = 0.3225), while above the threshold, the TyG index was positively associated with increased mortality risk (HR = 1.35, 95% CI: 1.20–1.52, *p* < 0.0001). However, the log‐likelihood ratio test for nonlinearity was not statistically significant (*p* = 0.112).

A more evident threshold effect was found for TyG‐BMI (Figure 2(b)), with an inflection point of 157.424. The hazard ratio was negligible below the threshold (HR = 0.02, 95% CI: 0.00–0.19, *p* = 0.0004), whereas a significant positive association was observed above it (HR = 1.22, 95% CI: 1.11–1.33, *p* < 0.0001), supported by a significant log‐likelihood ratio test (*p* = 0.002). Similar patterns were observed for TyG‐WC (Figure 2(c)) and TyG‐WHtR (Figure 2(d)), with respective inflection points at 1140.533 and 6.799. Above these thresholds, both indices were significantly associated with higher mortality risk (TyG‐WC: HR = 1.95, 95% CI: 1.34–2.82, *p* = 0.0004; TyG‐WHtR: HR = 2.08, 95% CI: 1.41–3.06, *p* = 0.0002). The log‐likelihood ratio tests for TyG‐WC and TyG‐WHtR indicated evidence of nonlinearity (*p* = 0.030 and *p* = 0.020, respectively). For TyG‐ABSI (Figure 2(e)), an upward trend in mortality risk was noted at higher values, with a significant association above the inflection point of 0.63 (HR = 1.39, 95% CI: 1.22–1.58, *p* < 0.0001). However, no significant evidence of nonlinearity was detected (*p* = 0.185).

### 3.4. Survival Patterns of HSV‐Positive Participants Across TyG Index Tertiles

**FIGURE 3:**
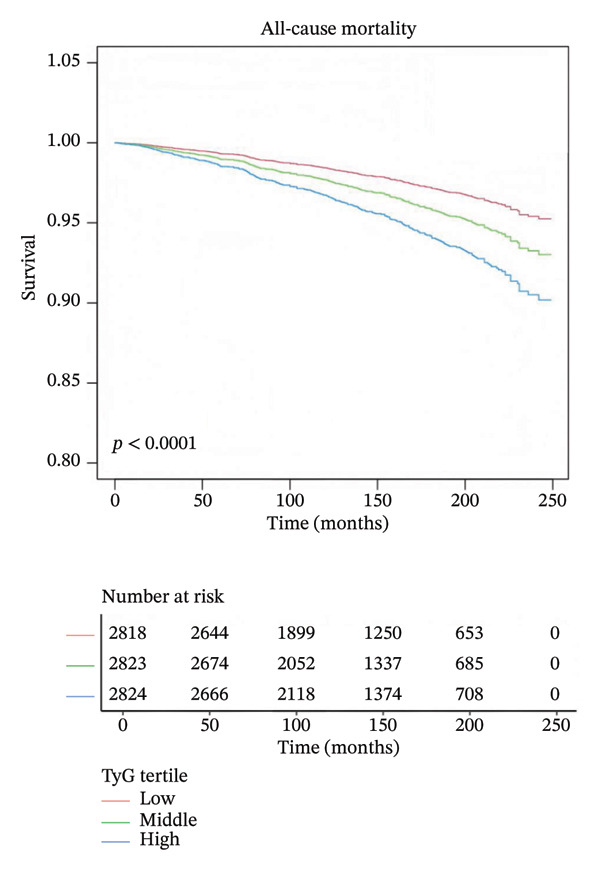
Kaplan–Meier curves for all‐cause mortality across tertiles of TyG index in HSV‐positive participants. Red line: low TyG tertile; green line: middle TyG tertile; blue line: high TyG tertile. Survival probability decreased with increasing TyG levels over the follow‐up period (in months).

Kaplan–Meier survival analysis was performed to examine the association between the TyG index and long‐term all‐cause mortality among HSV‐positive participants. As shown in Figure 3, participants in the highest TyG tertile exhibited the lowest survival probability over time, whereas those in the lowest tertile had the most favorable survival profile. The log‐rank test confirmed a significant difference in survival across TyG tertiles (*p* < 0.001).

### 3.5. Exploratory Analysis

As an exploratory analysis, GAMs were used to flexibly explore the potential dose–response associations between TyG‐related indices and cardiovascular mortality (Supporting Figure S1). After adjustment, the smooth curves indicated possible nonlinear patterns across all five indices (see Table 3).

**TABLE 3:** Baseline characteristics according to TyG index tertiles.

	Low (*N* = 2818)	Middle (*N* = 2823)	High (*N* = 2824)	*p* value
Age (years)	31.68 ± 9.57	34.12 ± 9.45	35.80 ± 8.88	< 0.001
Sex				< 0.001
Male	970 (34.42%)	1268 (44.92%)	1534 (54.32%)	
Female	1848 (65.58%)	1555 (55.08%)	1290 (45.68%)	
Height (cm)	166.66 ± 9.51	167.84 ± 9.94	168.14 ± 9.94	< 0.001
Weight (kg)	73.01 ± 18.89	81.44 ± 22.22	87.63 ± 21.39	< 0.001
BMI (kg/m^2^)	26.24 ± 6.33	28.88 ± 7.35	30.91 ± 6.66	< 0.001
Waist (cm)	88.06 ± 14.51	96.09 ± 15.76	102.93 ± 15.25	< 0.001
Waist‐to‐height ratio	0.53 ± 0.09	0.57 ± 0.10	0.61 ± 0.09	< 0.001
Triglyceride (mg/dL)	58.27 ± 14.69	103.11 ± 19.41	225.94 ± 169.11	< 0.001
Glucose (mg/dL)	5.07 ± 0.51	5.33 ± 0.64	6.25 ± 2.71	< 0.001
TyG	7.85 ± 0.28	8.48 ± 0.16	9.28 ± 0.49	< 0.001
TyG‐BMI	206.06 ± 51.15	244.80 ± 60.77	287.12 ± 64.86	< 0.001
TyG‐WC	691.64 ± 120.4	815.39 ± 136.17	956.30 ± 156.97	< 0.001
TyG‐WHTR	4.16 ± 0.74	4.87 ± 0.82	5.70 ± 0.93	< 0.001
TyG‐ABSI	0.61 ± 0.04	0.68 ± 0.04	0.75 ± 0.06	< 0.001
Ethnicity				< 0.001
Non‐Hispanic White	771 (27.36%)	937 (33.19%)	1013 (35.87%)	
Non‐Hispanic Black	987 (35.02%)	644 (22.81%)	398 (14.09%)	
Mexican American	565 (20.05%)	758 (26.85%)	903 (31.98%)	
Other Hispanic	240 (8.52%)	257 (9.10%)	294 (10.41%)	
Other Race	255 (9.05%)	227 (8.04%)	216 (7.65%)	
Marital status, *n* (%)				< 0.001
Married/living with partner	1354 (48.05%)	1635 (57.92%)	1824 (64.59%)	
Widowed/divorced/separated	309 (10.97%)	338 (11.97%)	381 (13.49%)	
Never married	966 (34.28%)	744 (26.35%)	532 (18.84%)	
Missing	189 (6.71%)	106 (3.75%)	87 (3.08%)	
Poverty income ratio				0.295
Poor	725 (25.73%)	710 (25.15%)	729 (25.81%)	
Nearly poor	668 (23.70%)	724 (25.65%)	750 (26.56%)	
Middle income	659 (23.39%)	662 (23.45%)	632 (22.38%)	
High income	540 (19.16%)	520 (18.42%)	489 (17.32%)	
Missing	226 (8.02%)	207 (7.33%)	224 (7.93%)	
Education level, *n* (%)				< 0.001
Below high school	192 (6.81%)	260 (9.21%)	373 (13.21%)	
High school	1242 (44.07%)	1288 (45.63%)	1344 (47.59%)	
Above high school	1382 (49.04%)	1274 (45.13%)	1103 (39.06%)	
Missing	2 (0.07%)	1 (0.04%)	4 (0.14%)	
Smoking status, *n* (%)				< 0.001
Never	1618 (57.42%)	1499 (53.10%)	1371 (48.55%)	
Former	310 (11.00%)	366 (12.96%)	514 (18.20%)	
Now	533 (18.91%)	742 (26.28%)	818 (28.97%)	
Missing	357 (12.67%)	216 (7.65%)	121 (4.28%)	
Alcohol use, *n* (%)				< 0.001
Never	337 (11.96%)	328 (11.62%)	315 (11.15%)	
Former	241 (8.55%)	290 (10.27%)	401 (14.20%)	
Mild	618 (21.93%)	630 (22.32%)	593 (21.00%)	
Moderate	462 (16.39%)	386 (13.67%)	345 (12.22%)	
Heavy	528 (18.74%)	742 (26.28%)	821 (29.07%)	
Missing	632 (22.43%)	447 (15.83%)	349 (12.36%)	
Total physical activity (MET/week)				< 0.001
< 600	748 (26.54%)	822 (29.12%)	796 (28.19%)	
≥ 600	1473 (52.27%)	1360 (48.18%)	1270 (44.97%)	
Missing	597 (21.19%)	641 (22.71%)	758 (26.84%)	
Hypertension				< 0.001
No	2440 (86.59%)	2264 (80.26%)	2023 (71.66%)	
Yes	378 (13.41%)	557 (19.74%)	800 (28.34%)	
Diabetes mellitus				< 0.001
No	2539 (93.11%)	2286 (85.97%)	1611 (63.23%)	
Yes	46 (1.69%)	101 (3.80%)	475 (18.64%)	
IFG	60 (2.20%)	132 (4.96%)	285 (11.19%)	
IGT	82 (3.01%)	140 (5.27%)	177 (6.95%)	
Hyperlipidemia				< 0.001
No	1802 (63.95%)	1044 (36.98%)	133 (4.71%)	
Yes	1016 (36.05%)	1779 (63.02%)	2691 (95.29%)	
Cardiovascular diseases				< 0.001
No	2341 (97.99%)	2526 (97.53%)	2593 (96.29%)	
Yes	48 (2.01%)	64 (2.47%)	100 (3.71%)	

*Note:* WHtR, waist‐to‐height ratio; TyG, triglyceride–glucose index.

Abbreviations: BMI, body mass index; IFG, impaired fasting glycemia; IGT, impaired glucose tolerance; MET, metabolic equivalent of task; WC, waist circumference.

## 4. Discussion

To our knowledge, this is the first study to examine associations between the TyG index and its obesity‐related derivatives with mortality in an HSV‐positive population. These findings extend previous observations from general and disease‐specific cohorts to a younger, infection‐defined group. One key finding emerged: Composite indices combining the TyG index with measures of adiposity showed robust and, in several cases, nonlinear associations with all‐cause mortality, often stronger than those observed for the TyG index alone.

The observed relationship between elevated TyG and mortality is biologically plausible. As a marker of IR, the TyG index reflects elevated triglycerides and glucose [24, 25], which contribute to vascular damage through mechanisms such as endothelial dysfunction and inflammation [26]. These results are consistent with previous studies in general and hypertensive populations, and our findings suggest that metabolic risk may manifest earlier in adulthood [27].

The threshold identified at TyG ≈7.9 suggests a range within which increases in TyG may not confer excess risk, while higher levels are associated with increased mortality. In our study, risk was elevated only above this cutoff, contrasting with findings in older or frail populations where very low TyG may reflect underlying illness. For example, a U‐shaped association between TyG‐BMI and mortality was observed in diabetic individuals [28], but not in our younger cohort, possibly due to fewer participants with low metabolic status.

Composite indices incorporating TyG and obesity measures provided additional insight. All four indices showed significant associations with mortality, with nonlinear relationships and clear inflection points. TyG‐BMI and TyG‐WC performed similarly to TyG alone. ABSI reflects abdominal shape independent of BMI, potentially identifying individuals with visceral adiposity and metabolic dysfunction [29, 30].

Focusing on an HSV‐positive population provides a specific epidemiological context in which to examine associations between IR‐related indices and mortality. HSV infection is highly prevalent and heterogeneous, and seropositivity reflects lifelong viral exposure rather than active disease or clinical manifestations [31]. Prior evidence suggests that chronic viral infections, including HSV, may be associated with low‐grade systemic inflammation and metabolic dysregulation, which could contextualize the relationship between IR markers and long‐term mortality risk at the population level [19, 32]. Importantly, HSV seropositivity in NHANES does not capture viral activity, reactivation frequency, or clinical symptoms, and our analysis does not evaluate effect modification by HSV infection. Accordingly, the present findings should be interpreted as population‐level associations rather than evidence of prognostic utility or causal effects. Restricting the analysis to HSV‐positive adults allows us to describe how TyG‐related indices relate to mortality within a defined chronic viral exposure background, without implying that HSV infection itself modifies or drives these associations.

Our findings are broadly consistent with prior studies conducted in the general population, which have reported positive associations between the TyG index or TyG‐related composite indices and all‐cause mortality [12, 33]. Several large cohort studies have shown that higher TyG levels are associated with increased mortality risk, with effect estimates generally in the range of 1.2–1.5 per unit or per category increase, and with evidence of nonlinear or threshold patterns, particularly for composite indices incorporating adiposity measures [7, 13].

In this context, the magnitude and shape of the associations observed in our HSV‐positive population are comparable to those reported in the general population but appear more pronounced for indices that integrate both IR and central adiposity. This pattern is in line with prior evidence suggesting that composite TyG‐related indices may better capture metabolic vulnerability than the TyG index alone [7, 13]. Similar observations have been reported in other chronic viral infection settings, most notably among people living with HIV, where IR and adiposity‐related markers have been linked to increased cardiometabolic and mortality risk [34–36]. Although the mechanisms and clinical contexts differ, these studies support the concept that chronic viral exposure may provide a background in which metabolic dysfunction has heightened epidemiological relevance. Importantly, our results should not be interpreted as evidence of a shared causal pathway across infections but rather as population‐level associations that align with patterns observed in other chronic infection contexts.

Given the observational nature of this study based on a cohort analysis of NHANES data, our findings reflect associations rather than causal effects. These findings underscore the relevance of metabolic monitoring in HSV‐positive individuals. Although HSV infection itself is often benign, elevated TyG and adiposity measures may help identify individuals at greater long‐term mortality risk. Lifestyle or medical strategies targeting metabolic health could be explored in future longitudinal research; however, whether modifying TyG levels can reduce risk remains to be determined.

In the multivariable models, baseline CVD and dyslipidemia were not included as covariates. Although both conditions were more prevalent among nonsurvivors, they may lie on the pathway linking IR to mortality, and adjustment for these factors could result in overadjustment and attenuation of the associations of interest. To assess the robustness of our findings, we conducted sensitivity analyses with additional adjustment for diabetes and hypertension (Supporting Figure S2). The associations between TyG‐related indices and all‐cause mortality remained generally consistent after this further adjustment, supporting the stability of the observed associations.

Several limitations should be acknowledged. First, the cross‐sectional assessment of exposures at baseline limits causal inference; observed associations may reflect reverse causality or residual confounding, despite adjustment for a range of covariates and exclusion of early deaths. Second, TyG and obesity‐related indices were measured only once at baseline, and dynamic changes in metabolic status during follow‐up could not be captured, potentially leading to misclassification or regression dilution bias. Third, although HSV‐positivity provided a novel population framework, generalizability to HSV‐negative individuals or those with other chronic infections may be limited. Fourth, we lacked data on inflammatory biomarkers, antiviral therapy, or detailed clinical comorbidities, which could further clarify the mechanisms linking TyG to mortality. Fifth, the number of cardiovascular deaths was relatively small, which limited statistical power for this secondary outcome and precluded detailed cause‐specific mortality analysis. Finally, unmeasured lifestyle factors or genetic predispositions may have influenced the observed associations.

Despite these limitations, this study has several strengths. It leverages nearly 2 decades of follow‐up in a well‐characterized, nationally based sample of younger adults, which is a population segment less studied in mortality research. The use of multiple TyG‐based indices allowed a comprehensive evaluation of metabolic risk dimensions. We employed advanced analytical methods to uncover nonlinear effects that would have been missed by simple linear assumptions. Additionally, our analysis controlled for a wide range of demographic and lifestyle factors, strengthening the validity of the observed associations.

## 5. Conclusion

Elevated levels of the TyG index and its obesity‐related derivatives, particularly TyG‐WHtR, were independently associated with increased all‐cause mortality in HSV‐positive adults. These findings may contribute to a better understanding of metabolic risk patterns in this population.

## Author Contributions

Jun Wei contributed to the conception and design of the study. Jun Wei and Jun Zhang were responsible for data analysis and interpretation. Yang Liu drafted the manuscript and critically revised it for important intellectual content.

## Funding

This research was supported by the Discipline Construction Fund of Jilin Medical University—Human Anatomy and Histology & Embryology (grant number: 1642022502).

## Disclosure

All authors read and approved the final version of the manuscript and agree to be accountable for all aspects of the work.

## Ethics Statement

This study is based on publicly available data from the National Health and Nutrition Examination Survey (NHANES), conducted by the National Center for Health Statistics (NCHS). All procedures involving human participants were approved by the NCHS Research Ethics Review Board, under Protocol #2005‐06 and Protocol #2011–17. Written informed consent was obtained from all participants at the time of original data collection.

## Conflicts of Interest

The authors declare no conflicts of interest.

## Supporting Information

Supporting Figure S1. Smooth curve fitting for TyG‐related indices and cardiovascular mortality.

## Supporting information


**Supporting Information** Additional supporting information can be found online in the Supporting Information section.

## Data Availability

The datasets generated and analyzed during the current study are available in the NHANES repository (https://wwwn.cdc.gov/nchs/nhanes/Default.aspx).
