# Supplementary material for: TyG Index and Obesity‐Related Measures in Relation to All‐Cause Mortality Among HSV‐Positive Adults
Source: Int J Endocrinol. 2026 Feb 26;2026:1608622. doi: 10.1155/ije/1608622 (PMC12936696; doi:10.1155/ije/1608622)
Supplement: Supplementary file 1 — Supporting Information Additional supporting information can be found online in the Supporting Information section. [file IJE-2026-1608622-s001.docx]

Supplementary Figure S1. Smooth curve fitting for TyG-related indices and cardiovascular mortality.


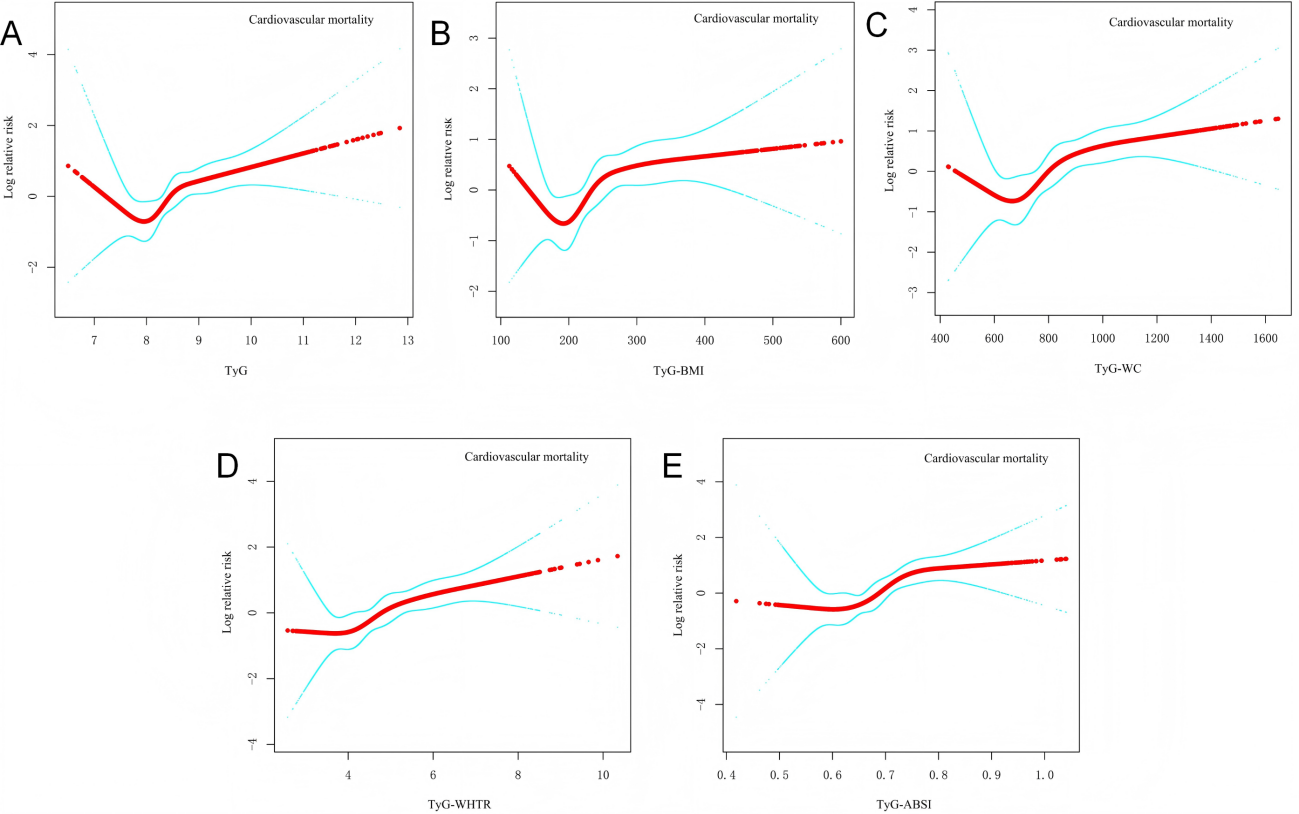


Smooth curve fitting of the associations between TyG-related indices and cardiovascular mortality. Panels show the dose–response relationships between cardiovascular mortality risk and **(A)** TyG index, **(B)** TyG-BMI index, **(C)** TyG-WC index, **(D)** TyG-WHtR index, and **(E)** TyG-ABSI index. Solid red lines represent the estimated log-relative risk derived from generalized additive models, and light blue shaded areas indicate the corresponding 95% confidence intervals. Models were adjusted for age (years), sex, ethnicity, marital status, poverty income ratio, education level, smoking status, alcohol use, and total physical activity (MET/week). **Abbreviations:** TyG, triglyceride–glucose index; ABSI, a body shape index; BMI, body mass index; WC, waist circumference; WHtR, waist-to-height ratio.

Supplementary Figure S2. Smooth curve fitting of the associations between TyG-related indices and all-cause mortality after additional adjustment for diabetes and hypertension.


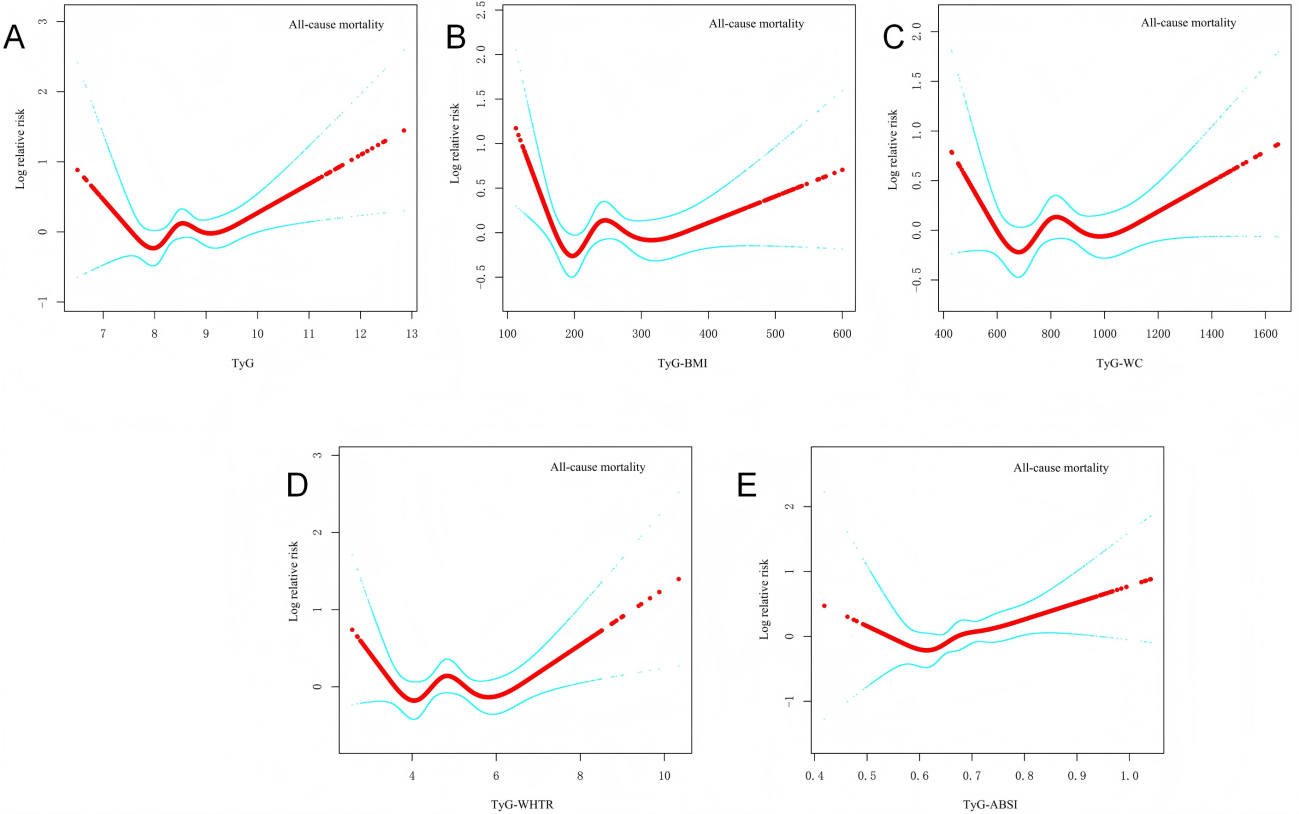


Panels show the dose–response relationships between all-cause mortality risk and (A) TyG index, (B) TyG-BMI, (C) TyG-WC, (D) TyG-WHtR, and (E) TyG-ABSI based on generalized additive models. Solid red lines represent the estimated log-relative risk, and light blue shaded areas indicate the corresponding 95% confidence intervals. Models were adjusted for age (years), sex, ethnicity, marital status, poverty income ratio, education level, smoking status, alcohol use, total physical activity (MET/week), **and additionally for diabetes and hypertension**. **Abbreviations:** TyG, triglyceride–glucose index; BMI, body mass index; WC, waist circumference; WHtR, waist-to-height ratio; ABSI, a body shape index.
